# The Role of CD276 in Cancers

**DOI:** 10.3389/fonc.2021.654684

**Published:** 2021-03-26

**Authors:** Shengzhuo Liu, Jiayu Liang, Zhihong Liu, Chi Zhang, Yang Wang, Alice Helen Watson, Chuan Zhou, Fan Zhang, Kan Wu, Fuxun Zhang, Yiping Lu, Xianding Wang

**Affiliations:** ^1^ Department of Urology, Institute of Urology, West China Hospital, Sichuan University, Chengdu, China; ^2^ William Harvey Research Institute, Queen Mary University of London, London, United Kingdom; ^3^ Laboratory of Aging Research and Cancer Drug Target, State Key Laboratory of Biotherapy, National Clinical Research Center for Geriatrics, West China Hospital, Sichuan University, Chengdu, China; ^4^ Clinical Science and Services, Royal Veterinary College, University of London, London, United Kingdom

**Keywords:** CD276, B7-H3, therapeutic target, tumor, progression

## Abstract

**Objective:**

Aberrant expression of the immune checkpoint molecule, CD276, also known as B7-H3, is associated with tumorigenesis. In this review, we aim to comprehensively describe the role of CD276 in malignancies and its potential therapeutic effect.

**Data Sources:**

Database including PubMed, EMbase, Cochrane Library, CNKI, and Clinical Trails.gov were searched for eligible studies and reviews. Study selection: Original studies and review articles on the topic of CD276 in tumors were retrieved.

**Results:**

CD276 is an immune checkpoint molecule in the epithelial mesenchymal transition (EMT) pathway. In this review, we evaluated the available evidence on the expression and regulation of CD276. We also assessed the role of CD276 within the immune micro-environment, effect on tumor progression, and the potential therapeutic effect of CD276 targeted therapy for malignancies.

**Conclusion:**

CD276 plays an essential role in cell proliferation, invasion, and migration in malignancies. Results from most recent studies indicate CD276 could be a promising therapeutic target for malignant tumors.

## Introduction

Immune Checkpoint Therapy (ICT) is a novel treatment for malignancy which shows great efficacy by enhancing anti-tumor immune response of T cells. The B7 family, which is important in regulating T cell immune response, has been highlighted for its potential therapeutic effect on cancers. B7 family members, including B7-H1 (PD-L1/CD274), B7-DC (PD-L2/CD273), B7-H2 (ICOSL), B7-H3 (CD276), B7-H4 (B7S1/B7x/Vtcn1), B7-H5 (VISTA/GI24/Dies/PD-1H), B7-H6 (NCR3LG1), B7-H7 (HHLA2), B7.1 (CD80), and B7.2 (CD86), could be categorized into (a) co-stimulatory, (b) co-inhibitory, or (c) mixed co-stimulatory and co-inhibitory subtype according to how they influence T cell activation (1).For example, PD-L1/B7-H1 is the inhibitory ligand of PD-1 death receptor and serves as an immune regulator by reducing T cell proliferation. B7-H3 (CD276), as a member of B7 family, was first introduced by Chapoval et al. in 2001 (2). Recent studies found a newly emergent immune checkpoint molecule, CD276, which serves as a T cell inhibitor to promote tumor proliferation and invasion, rather than CD276 acting as a T cell stimulatory molecule as previously described. Our study revealed that CD276 is an EMT pathway associated immune checkpoint. This review summarizes the available evidence to the expression of CD276 in tumors and its regulation mechanism, CD276 in immune micro-environment, effect on tumor progression, and its potential therapeutic effect for malignancies.

## Expression and Regulation of CD276 in Tumor

### Expression Levels of CD276 in Tumors

CD276 mRNAs are commonly expressed at a relatively low level in most normal tissues. In contrast, in malignant tumor tissues CD276 expression levels are up-regulated. Moreover, higher expression levels of CD276 has been found to be correlated with poorer prognosis in cancer patients. Relevant studies have been conducted in a wide range of tumor types, including cancers of the bladder, breast, cervix, colorectal, esophageal, renal, hepatic, lung, ovarian, pancreatic, prostate, biliary, oral squamous cell carcinoma, intrauterine membranous cancer, squamous cell carcinoma, gastric cancer, glioma, melanoma, and adrenal malignancies (3–25) (shown as **Table 1**).

**Table 1:** The expression of CD276 in tumors and its relationship with pathological features.

Tumor type	Expression	Clinical significance	Reference
Bladder cancer	High	Not reported	(3)
Endometrial cancer	High	Associated with poor prognosis and TNM stages	(4)
Pancreatic cancer	High	Associated with lymph node migration, tumor size, TNM stages, and cancer recurrence	(5, 6)
Cervical cancer	High	Associated with poor prognosis and tumor size	(7)
Breast cancer	High	Associated with poor prognosis, tumor size, TNM stages, lymph node migration, and cancer recurrence	(8, 9)
Intrahepatic cholangiocarcinoma	High	Associated with poor prognosis, lymph node migration, tumor’s progression, and cancer recurrence	(10)
Colorectal cancer	High (membrane, cytoplasm, and nuclei)	Associated with poor prognosis, TNM stages, lymph node migration, and cancer recurrence	(11)
Ovarian cancer	High (membrane, cytoplasm, and tumor endothelium)	Associated with poor prognosis and TNM stages	(12)
Glioma	High	Associated with TNM stages	(13)
Melanoma	High	Associated with poor prognosis and TNM stages	(14)
Lung cancer	High	Associated with poor prognosis, TNM stages, lymph node migration, and cancer Recurrence	(15, 16)
Liver cancer	High	Associated with poor prognosis, lymph node migration, tumor’s progression, and TNM stages	(17)
Prostatic cancer	High	Associated with poor prognosis, lymph node migration, tumor’s progression, TNM stages, and cancer recurrence	(18, 19)
Oral squamous cell carcinoma	High	Associated with poor prognosis, tumor size, and TNM stages	(20)
Kidney cancer	High (tumor vessels and tumor cells)	Associated with poor prognosis, tumor size, and TNM stages	(21)
Pancreatic cancer	High	Positively correlated to the prognosis of patients	(22)
Gastric cancer	High	Positively correlated to the prognosis of patients	(24)
Adrenocortical carcinoma	High (membrane, cytoplasm, and tumor vessels)	Negatively correlated to the prognosis of patients Associated with tumor size and TNM stages	(25)

Genitourinary tumors provide a good example of elevated CD276 expression in tumor tissue. Xylinas et al. demonstrated that expression levels of PD-1 and CD276 were significantly higher in bladder urothelial carcinoma tissue when compared with adjacent normal tissues (3). In another study, Benzon et al. explored the association between CD276 expression and clinicopathologic variables. CD276 expression level was found to be positively correlated with Gleason score, tumor stage, castration resistance, and metastasis. They also performed a gene set enrichment analysis and results indicated CD276 was associated with androgen signaling as well as immune regulatory pathways (19). Another study conducted on a cohort of patients treated for clear cell renal cell carcinoma (ccRCC) reported that CD276 expression was detected in 17% of tumor cells and 95% of tumor vasculature. Expression of CD276 in ccRCC specimens is correlated with adverse clinicopathologic outcomes (tumor size, tumor stage, nuclear grading, coagulative tumor necrosis, and sarcomatoid differentiation). In multivariable analysis, the presence of CD276 in tumor cells or diffusely within tumor vasculature was significantly associated with an increased risk of death from ccRCC (21).

Abnormal CD276 expression has also been observed in rare tumors with a high degree of malignancy. Adrenocortical carcinoma (ACC) is characterized as a malignant adrenal tumor with a high recurrence rate and a short disease-free survival, and 91.7% (44/48) of ACCs expressed cytoplasmic and membranous CD276 in neoplastic or tumor associated vascular cells. CD276 expression in tumor blood vessels was significantly positively correlated with local invasion (higher T stage) and with advanced ENSAT stage (European Network for the Study of Adrenal Tumors). Cytoplasmic CD276 expression is negatively correlated with both the overall survival and disease-free survival of ACC patients. Gene set enrichment analysis demonstrated that CD276 expression is closely related to immune regulation and malignant biological behavior of ACC (24).

**Figure 1:**
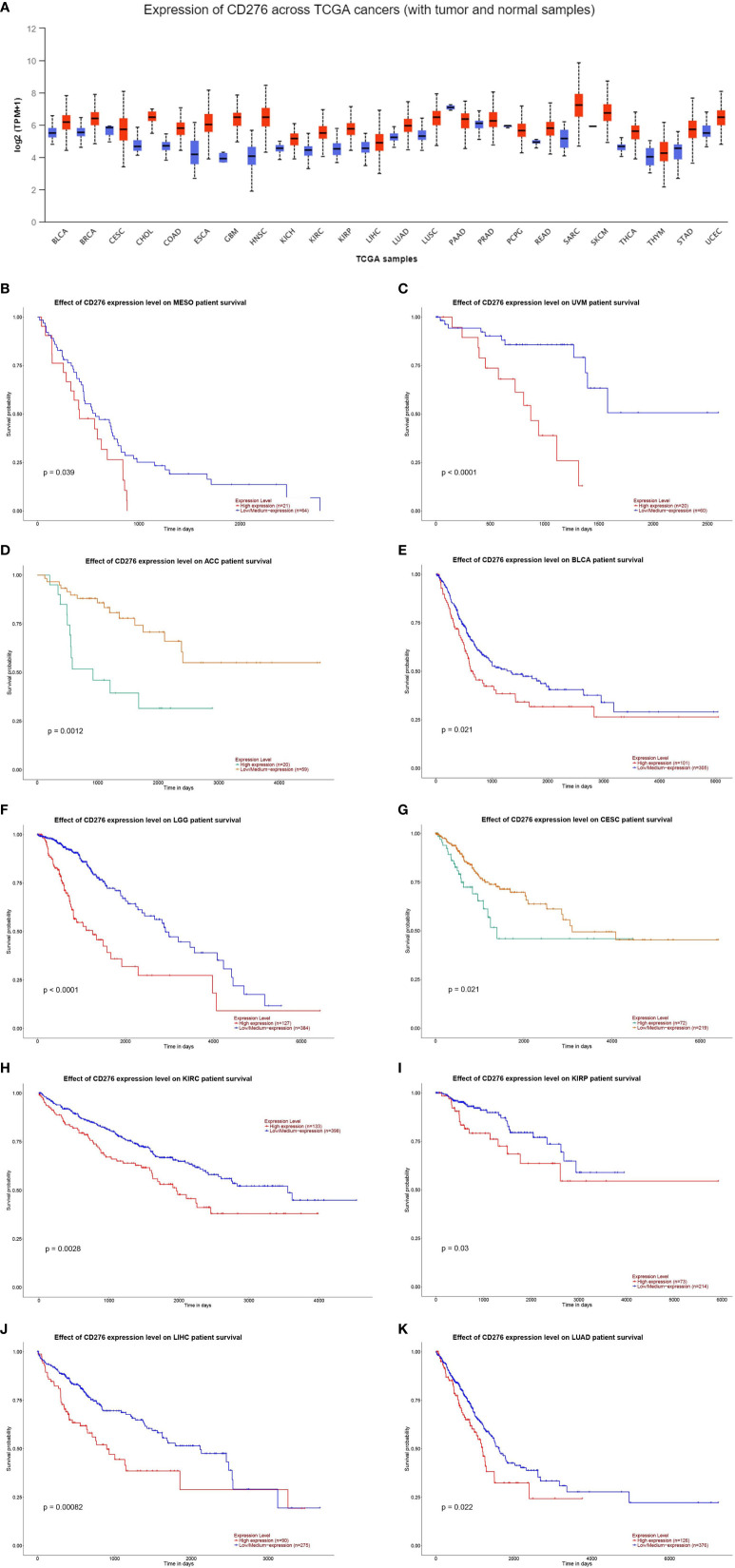
**(A)**: CD276 expression level in cancer and corresponding normal tissues. **(B–K)**: The correlation of CD276 expression with survival in cancer patients (p < 0.05).

To give a more intuitive aspect of the expression level of CD276 in malignancies, we performed a pan-cancer analysis to evaluate CD276 expression level in multiple cancers on UALCAN database (http://ualcan.path.uab.edu/index.html). UALCAN is an online web-server for facilitating tumor subgroup gene expression and survival analyses based on TCGA RNA sequencing data and clinical data. CD276 expression level in cancer and corresponding normal tissues is shown **Figure 1A**. We also explored the correlation of CD276 expression with survival and found CD276 expression is significantly correlated with survival probability in a wide range of cancer types (p < 0.05) (**Figures 1B–K**).

### Regulatory Mechanisms of CD276

Although CD276 mRNA expression is detected in most human tissues, CD276 protein expression is not ubiquitous (26–28). The difference between CD276 mRNA and protein expression suggests that post-transcriptional regulation mechanisms may cause the discrepancy between transcription and translation (29, 30). Previously our understanding of the molecular mechanisms behind the regulation of CD276 expression was limited. Woong et al. (30) performed an *in vivo* study and found that in mouse dendritic cells CD276 expression could be enhanced by interferon-γ (IFN-γ) and suppressed by interleukin 4 (IL-4). They concluded that CD276 was a negative regulator of T cell function preferentially affecting type-1 T helper cell responses (30). CD4^+^CD25 regulatory T cells (Tregs) have immunosuppressive effects and can inhibit the proliferation of effector T cells both *in vivo* and *in vitro* (31). Myeloid dendritic cells co-cultured with regulatory T cells are able to induce up-regulation of CD276 expression, thereby affecting the maturation of dendritic cells (32). MicroRNAs (miRNAs) can also regulate the expression of CD276 through direct interaction with the 3′-UTR of CD276 mRNA and decreasing the level of protein translation (33, 34). miRNA-29 is highly expressed in normal tissues and poorly expressed in a variety of solid tumors, which is negatively correlated with CD276 expression. Luciferase reporter gene analysis shows a conserved miR-29-targeting site of CD276 in the 3 ‘UTR region (35, 36). In the study of CD276 in cutaneous melanoma, researchers found that MiR-29c expression could inversely regulate CD276 expression (14). However, the mechanism regulating the downstream signaling pathway by CD276 remains to be elucidated. Immunoglobulin-like transcript 4 (ILT4) is another molecule regulated by CD256. ILT4 belongs to the immunoglobulin-like super family that inhibit immune cell function (37). Research indicates that ILT4 mediated activation of PI3K/AKT/mTOR pathway increases CD276 expression in non-small cell lung cancer (NSCLC) (38). PDZ-binding kinase (PBK), whose expression is associated with immune infiltration in nasopharyngeal carcinoma (NPC), also plays an important role in CD276 expression regulation. In a recent study conducted by Wang et al., they demonstrated that the phosphorylation level of MSL1 was enhanced in PBK-overexpression cells and meanwhile the expression of CD276 was up-regulated. The up-regulation of CD276 following PBK-overexpression could be reversed by MSL1 depletion. Mechanistically, MSL1 serves as a mediator in the PBK-MSL1-CD276 axis (39). Overall, there are many intertwined factors contributing to the regulation of CD276 expression, which result in the observed relatively low level expression in normal tissue and elevated expression in tumor tissue. Further investigations are required to develop our understanding of CD276 expression regulation.

### Potential Receptors of CD276

The immune function of the majority of the B7 family is mediated by a variety of known receptors. For example, B7-1 or B7-2 bind to CD28 and CTLA-4, PD-L1 (B7-H1) or PD-L2 (B7-DC) bind to PD-1, and B7-H6 binds to NKp30 (40, 41). However, the receptor for CD276 has not been verified. According to Hashiguchi’s findings, CD276 receptor may be expressed in a Trem-like transcript 2 (TLT-2 or TREML2) (42, 43). After introduction of TLT-2 into T cells, stimulating with CD276 resulted in increased production of interleukin-2 (IL-2) and IFN-γ (37, 42). CD276 was initially mooted as a ligand for TREM receptor family. However, subsequent studies showed contradictory results. A study by the Leitner group found no evidence for an interaction between CD276 and TREML2 in mouse or human cells (44). Furthermore, size exclusion chromatography (SEC) and surface plasmon resonance experiments demonstrated that murine CD276 and TLT-2 do not bind one another (45). Those contradictory results suggested that TLT-2 is unlikely to be a receptor for CD76, and an alternative receptor interacts with CD276 to mediate inhibitory immune responses.

In 2019, Husain et al. (46) implemented a new platform (conditioned medium AlphaScreen) to sensitively detect receptor-ligand interactions at high throughput. With the help of the technology, Husain identified interleukin receptor IL20RA as a receptor for the checkpoint inhibitor CD276. Further confirmatory studies about CD276 receptors are being conducted.

## CD276 and Immune Checkpoint Signals

CD276 was initially described as a co-stimulatory molecule for T cell activation (2). But subsequent research revealed that CD276 had a strong immunosuppressive effect, and could inhibit the proliferation of T cells (47). Furthermore, up-regulated CD276 promotes immune escaping of tumor cells, reducing the secretion of IFN-γ, tumor necrosis factor-alpha (TNF-α), and other cytokines (48). Though a number of studies reported contradictory results and the receptor-mediated mechanism has not been clearly elucidated, mounting evidence demonstrates that CD276 has synergetic effects with other immune checkpoints.

### CD276 and PD-1

Programmed Cell Death-1 (PD-1) is an immunosuppressive receptor, first reported in 1992 (49). PD-1, also known as B7H1 and CD279, is encoded by PDCD1 gene on chromosome 2. PD-1 is a member of CD28 super family and is located on T cell and B cell surfaces. CD28 family members play an important role in the regulation of T cell activity through interaction with ligands and transmission of inhibitory signals (50). PD-1 can interact with PD-L1 (B7-H1) and PD-L2 (B7-DC), thereby down-regulating the activity of effector T cells in normal tissues and tumors (51, 52).

PD-1 is one of the checkpoint receptors, which is tightly associated with immune escaping of tumor cells. Such a characteristic renders PD-1/PD-L1/PD-L2 as a significant target for immunotherapy, which has been applied to cancer therapy and promising results were reported. Even so, PD-1/PD-L1/PD-L2 molecules are still only a small part of immunological checkpoints with therapeutic values. There are more potential immune checkpoints which may be used as therapeutic targets. PD-1 and CD276 are both members of B7/CD28 family and had similar effects on the tumor growth micro-environment. The binding of CD276/PD-1 and corresponding receptors inhibits the proliferation of T cells and the secretion of IFN-γ, TNF-α, and other cytokines, exerting synergistic effect with PD-1/PD-L1/PD-L2 (47, 48). Combination therapy with anti-CD276 antibody and anti-PD1/PD-L1 antibody for tumor treatment is another hot topic of research. In a recent study, it was reported that Photodynamic therapy (PDT) combined with PD1/PD-L1 and CD276 antibody respectively successfully improves the tumor suppressive blocking effect of PD-1/PD-L1 (53).

### Relationship Between CD276 and CTLA4

Another promising molecule for immune regulation is CTLA4 (CD152). CTLA4 is an intracellular glycoprotein that acts as a functional inhibitor of T cell responses (54). Mechanically, on the surface of dendritic antigen presenting cells, CD80/CD86 binds to CD28 on adjacent T cells, thereby activating T cells and producing cytokines. This interactive process is involved in multiple acute or chronic inflammatory diseases, such as atherosclerosis (55), autoimmune diseases (56), and malignant tumors (57). CTLA4 is a homologue of CD28, which has stronger binding affinity than CD28. CTLA4 can attenuate T cell activation once combined with antigen-presenting cells (APC) (58). Moreover, CTLA4 can reduce the contractive time between T cells and antigen-presenting cells. Therefore, an immunosuppressive microenvironment such as that mediated by CTLA4 can reduce the CD28-CD80/CD86 interaction time, and thus reduce T cell activation (59).

Based on the information above, CD276 inhibits T cell activation and has synergistic effects with CTLA4. Leitner et al. believe that CD276 exerts its inhibitory effect on T cells by inhibiting IL-2 secretion. Exogenous supplementation with IL-2 can reverse this effect, suggesting that CD276 acts as an upstream regulatory molecule for IL-2 (44). CTLA4 is expressed in normal tissues, normal immune cells, and tumor cells simultaneously. Compared to CTLA4 (60), CD276 is only expressed in tumors and tumor-related cells. Therefore, CD276 has higher specificity for tumor tissues.

## Role of CD276 in Tumorigenesis

CD276 can directly participate in the regulation of immunity and various malignant behaviors of tumor cells.

### CD276 in Tumor Cells

CD276 is involved in the regulation of proliferation, apoptosis, invasion, cell cycle, cell differentiation, cell autophagy, and epithelial-mesenchymal transition. To examine the role of CD276 in metabolic reprogramming of cancer cells, Lim et al. (61) conducted a study and found that tumor cells expressing CD276 significantly increase glucose uptake and lactate production both *in vivo* and *in vitro*. Thus, even in aerobic environments, cancer cells still produce energy by metabolizing glucose into lactic acid (anaerobic glycolysis), which is called Warburg effect and this is promoted by CD276. CD276 also increases the expression level of HIF-1α, as well as the expression of its downstream signaling molecules and the key enzymes in the glycolysis pathway (LDHA and PDK1). Furthermore, it is reported that CD276 promotes the stability of active oxygen-dependent HIF-1α by inhibiting the stress-activated transcriptional factor (Nrf2) and its targets (antioxidants SOD1, SOD2, and PRX3). Finally, a metabolic imaging experiment further confirmed that CD276 enhances the glucose uptake by tumor cells, thereby promoting tumor growth in a mouse breast cancer xenograft model. These findings provided a new perspective of immunomodulatory B7 family on tumor progression.

CD276 participates in tumor metastasis. The ability of melanoma cells to invade the gel decreases by silencing CD276 using shRNA. *In vivo*, silencing CD276 with shRNA prolongs the asymptomatic survival period. CD276 shRNA inhibits the expression of metastasis-related proteins in melanoma cells, such as matrix metalloproteinase MMP-2, tissue inhibitors of metalloproteinase (TIMP1 and TIMP2), signal transduction and transcription activator (STAT3), and IL-8 (62). Kang et al. suggest that CD276 determines the malignancy and invasive ability of liver cancers through the JAK3/STAT3/SLUG signaling pathway involving MMP2. The expression of epithelial-derived marker E-cadherin is down-regulated in epithelial-mesenchymal transition (EMT). The expression of E-cadherin in CD276-knockout liver cancer cells is increased significantly suggesting that CD276 plays a key role in EMT, which is enables the metastasis of liver cancer cells (63).

There is a correlation between the expression of MYC and CD276. Knocking out CD276 regulates the differentiation of malignant gliomas by increasing/decreasing expression of MYC, which causes silencing of SMAD6 (TGF-β pathway inhibitor) and enhanced SMAD4 expression (64). *In vivo*, CD276 knockout decreases the expression of MYC and inhibits tumor growth (64). In another study, down-regulation of CD276 renders the resistance of gastric cancer cells to radiotherapy by regulating apoptosis, cell cycle progression and DNA double-strand breaks. Furthermore, CD276 regulates the baseline level of autophagy. Adding rapamycin to CD276-up-regulated cells increases the baseline level of autophagy and enhances the sensitivity of radiotherapy (65).

### The Role of CD276 in Tumor Vessels

Nutrition is essential for tumor cell proliferation. New blood vessels are required in tumors to ensure substrate provision to cells distant from the normal vasculature. Normal tissue blood supply cannot guarantee the stable support of tumors. Therefore, angiogenesis markers are considered to be another characteristic of tumors (66). Angiogenesis inhibitors are used to suppress tumor progression. Researchers have demonstrated that tumors generate numerous new blood vessels in glioblastoma models (67). In addition, by selectively targeting endothelial cells produced by tumor stem cells, the researchers found that the number of tumor cells was significantly reduced and degraded. This phenomenon suggests that anti-angiogenesis is a potential target for anti-tumor therapy (68).

Recently, a number of studies have shown that CD276 is expressed in tumor vascular endothelial cells, suggesting that CD276 is related to tumor angiogenesis. It is reported that CD276 is expressed in the vascular system of renal tumors (21). Positive expression of CD276 is also observed in gastric adenocarcinoma blood vessels (24). CD276 is widely observed in vascular endothelial cells of colon, lung, and breast cancers (25). Importantly, CD276 is present only in the pathological blood vessels and is absent in physiological blood vessels. In addition to its expression in the tumor vascular system, CD276 also affects the tumor microenvironment through microvessels in a soluble form (sB7-H3). ELISA analysis showed that the expression level of sB7-H3 is elevated in the supernatant of pancreatic cancer cell culture, indicating that pancreatic cancer cells release sB7-H3 into the extracellular medium. Next, the researchers exposed pancreatic cancer cells to sB7-H3, finding that sB7-H3 significantly increased the migration and invasion of cancer cells (69). Furthermore, Lai et al. (70) studied the role of CD276 in angiogenesis using human umbilical vein endothelial cells (HUVECs). *In vitro* and *in vivo* experiments demonstrated that CD276 promotes the secretion of vascular endothelial growth factor (VEGF), which further promotes the formation of blood vessels. CD276 could also activate NF-κB signaling through a TLR-4-dependent mechanism, and this could cause subsequent up-regulation of VEGF and IL-8. Elevated expression levels of VEGF and IL-8 further promote tumor invasion and angiogenesis (71). In general, CD276s role in tumor angiogenesis should be a topic of concern and is worthy of further research. It is expected to be a new target for anti-tumor angiogenesis.

## CD276 and Immunotherapy

**Figure 2:**
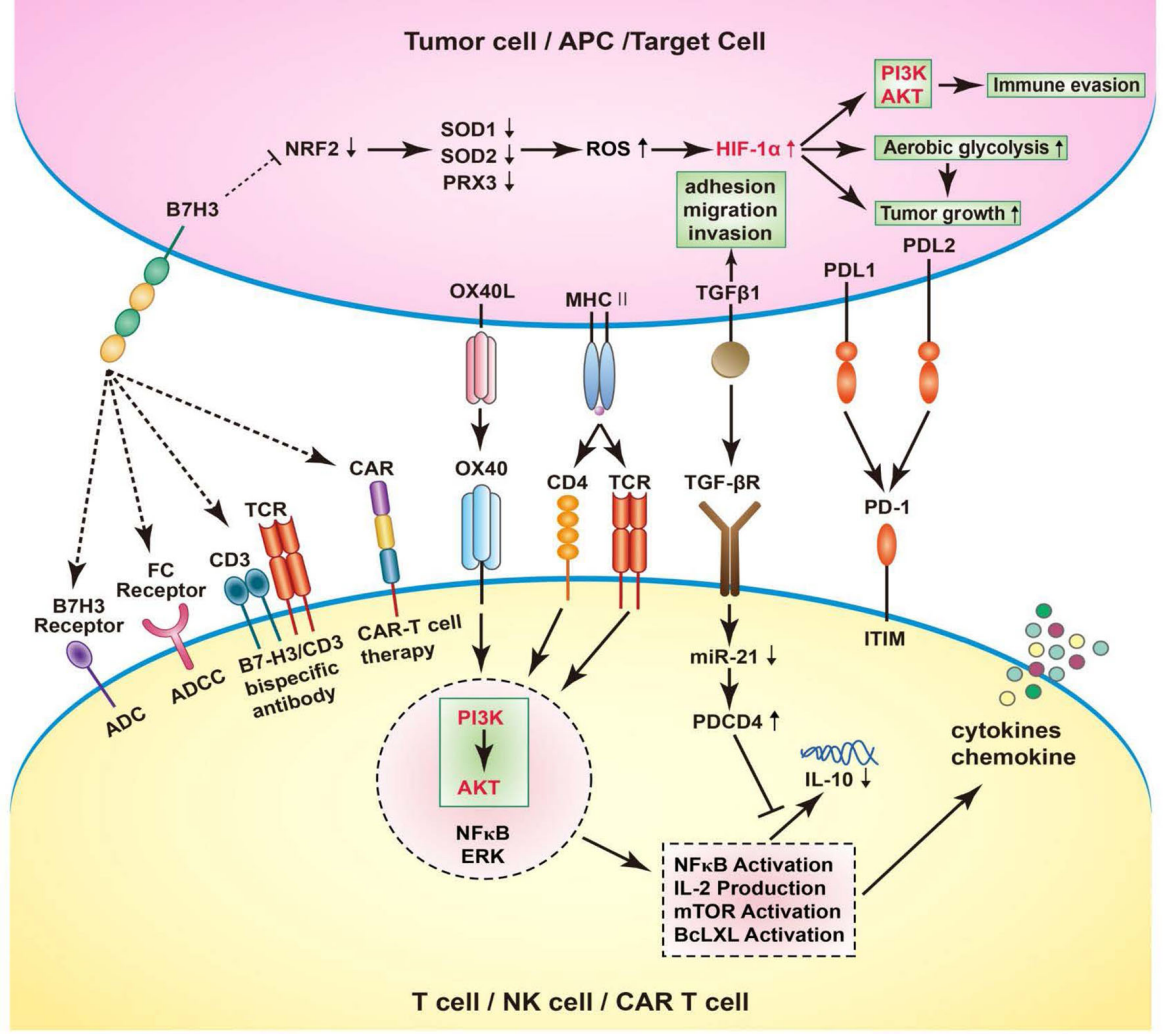
B7-H3 increases ROS and HIF-1α through B7-H3-induced Nrf2 suppression, SOD1, SOD2, and PRX3 reduction, thus promote aerobic glycolysis in cancer cells, leading to tumor growth. Human cancer immunotherapy strategies targeting B7-H3 including blockade of B7-H3 with blocking monoclonal antibodies (mAbs); B7-H3 specific antibody-dependent cell-mediated cytotoxicity (ADCC); CD3/B7-H3 bispecific antibodies; Small molecule inhibitors; Engineered chimeric antigen receptor (CAR) T cells; combination with other therapies. In T cells, OX40 engagement by OX40L forms a signaling complex with a number of established pro-inflammatory mediators, including AKT, PI3K, NFkB, ERK. Through the PI3K/AKT signaling pathway, the downstream signatures were activated, such as NFkB activation, IL-2 production, mTOR activation, BcLXL activation. As a result, NFkB activation stimulates the expression of chemokines and cytokines, including IL-10. Interestingly, the activation of TGFB1 receptor could simultaneously suppress the maturation of miR-21 and enhance PDCD4 levels. Consequently, the translation of the anti-inflammatory cytokine IL-10 is inhibited. In RCC cells, the TGFBI could participate in the adhesion, migration, and invasion, depending on the inactivation of VHL.

High hopes have been invested in immunotherapy for treatment of tumors in recent years. Immune checkpoints inhibitors (ICIs) targeting PD-1/PD-L1, CTLA-4 have been brought into clinical application and great efficacy was shown for numerous malignant tumors. As CD276 expression level was observed to be correlated to tumor growth, invasion, and metastasis, CD276 could be a potential target for immunotherapy. In previous studies, CD276 targeted therapies including monoclonal antibodies blocking CD276, CD276 specific antibody-dependent cell-mediated cytotoxicity (ADCC), antibody drug conjugates (ADC); bispecific antibodies against CD276/CD3, CD276–specific small–molecule inhibitor, and chimeric antigen receptor (CAR) T-cell therapy have been discussed (1, 34, 72, 73). In this review, we investigated the mechanisms underlying therapies targeting the immune checkpoint CD276 (**Figure 2**).

### CD276 and Gene Therapy

Drug vectors targeting the expression of CD276 may be an ideal therapeutic approach. Lupu et al. (74) injected the adenovirus vector expressing mB7-H3CD276 into colon tumors resulting in reduction of the primary tumor volume and secondary metastases. Yang et al. (75) demonstrated that transfecting CD276 plasmids into oral squamous cell carcinomas simultaneously enhances the proliferation of tumor cells, production of interferon, and the activity of cytotoxic T cells. Vasostatin is an effective inhibitor of angiogenesis. The combination of vasodilator and CD276-pcDNA3.1 inhibits tumor angiogenesis and promotes leukocyte infiltration in hepatocellular carcinoma (HCC). Notably, the combination is more effective than a single drug (76). Though promising CD276 is as a potential gene target in anti-tumor therapy, the negative effects of CD276 regulation should be considered before the clinical transformation. In addition, the best mechanism of drug delivery remains to be determined.

### Inhibitory Treatment With CD276 Antibody

To date, anti-CD276 antibodies have been developed, tested in animals, and are in clinical trials as immunotherapy agents. Xu et al. (36) confirmed that the antigen of monoclonal antibody 8H9 is 4Ig-B7-H3 (CD276). After CD276 is blocked with 8H9, anti-tumor effects are witnessed. When 8H9 is further combined with miR-29, the anti-tumor immune response is further enhanced. MJ18, another anti-CD276 monoclonal antibody, significantly promotes the infiltration of CD8+ T cells and inhibits the growth of pancreatic tumors (6). Loo et al. (77) synthesized MGA271, an Fc-engineered monoclonal antibody. MGA271 inhibition of CD276 in RCC and bladder cancer in tumor xenograft models resulted in inhibition of tumor growth. Currently, 8H9 and MGA271 are undergoing clinical trials (shown as **Table 2**).

**Table 2:** CD276 related clinical trials.

Condition or disease	Status
Relapsed and refractory neuroblastoma (NB)	Recruiting
CD276 positive solid tumor	Recruiting
Advanced CD276 positive solid tumor	Recruiting
Relapsed / refractory Acute Myeloid Leukemia	Recruiting
Central nervous system locoregional adoptive therapy	Recruiting
Recurrent Glioblastoma / Refractory Glioblastoma	Recruiting
Recurrent Glioblastoma / Refractory Glioblastoma	Recruiting
Neuroblastoma, Rhabdomyosarcoma, Osteosarcoma, Ewing Sarcoma, Wilms Tumor, Desmoplastic Small Round Cell Tumor	Completed
Advanced Solid Tumor, Metastatic Castrate Resistant Prostate Cancer, Non-small Cell Lung Cancer, Triple Negative Breast Cancer	Recruiting
Melanoma, Head and Neck Cancer, Non-small Cell Lung Cancer, Urethelial Carcinoma	Active, not recruiting
Advanced Solid Tumors	Active, not recruiting
Melanoma, Non-small Cell Lung Cancer	Completed
Melanoma	Terminated
Prostate Cancer	Active, not recruiting
Prostate Cancer, Melanoma, Renal Cell Carcinoma, Triple-negative Breast Cancer, Head and Neck Cancer, Bladder Cancer, Non-small Cell Lung Cancer	Completed

MGD009: B7-H3 x CD3 DART protein, is also known as orlotamab.

MGC018: Anti-B7-H3 antibody drug conjugate.

MGA012: Anti-PD-1 antibody, is also known as INCMGA00012.

MGA271: is also known as enoblituzumab.

MAb 376.96 is another anti-CD276 monoclonal antibody that recognizes the expression of CD276 epitopes on differentiated ovarian cancer cells and chemotherapy-resistant cancer cells (78). *In vitro* studies showed that mAb 376.96 combined with the tyrosine kinase inhibitor sunitinib significantly inhibits tumor growth. Ma et al. (48) constructed an anti-CD3 and anti-CD276 antibody (B7-H3Bi-Ab) targeting both T cell receptors and tumor-related antigens. B7-H3Bi-Ab bound to activated T-cells (ATC) significantly enhanced production of IFN-γ, TNF-α, and IL-2. Increased cytotoxicity mediated by B7-H3Bi-Ab is shown in different human tumor cell lines and in primary cells isolated from patients with breast and lung cancer. *In vivo*, experiments showed that, compared with ordinary activated T cells, B7-H3Bi-Ab assembled ATCs significantly inhibited tumor growth and prolonged survival (48). Seaman et al. reported that CD276-targeted antibody conjugated with drugs acting on tumor cells and tumor blood vessels simultaneously. In addition, m276-PBD dimer has shown extensive tumor killing effects and anti-metastasis activity *in vivo* (25). In a recent study conducted by Nathan et al. (79) a new CD276 targeting ADC m276-SL-PBD was introduced and its activity was explored in both pediatric solid malignancy patient-derived xenograft (PDX) and cell line-derived xenograft (CDX) models. Significant anti-tumor activity of m276-SL-PBD was observed in PDX models of solid tumors including Ewing sarcoma, rhabdomyosarcoma, Wilms tumors, osteosarcoma, and neuroblastoma. Another anti-CD276 ADC, MGC018 also shows antitumor activity across a range of human tumor xenografts (80).

Results of these studies suggest that CD276-targeted inhibitors may be an exciting immunotherapy strategy in the future.

### CD276-Chimeric Antigen Receptor (CAR) T Cell Therapy

Chimeric antigen receptor (CARs) T cell therapy is popular in immunotherapy. The objective response rate of CAR-T cell therapy in patients with hematologic malignancy is high. In solid tumors, due to the lack of tumor-specific antigens and the immunosuppressive effect of tumor microenvironment, there are some obstacles for CAR T cells to overcome. The gene module programming technology can improve the efficacy and specificity of CAR T, which is expected to target solid tumors for CAR T cells.

In 2019, Du et al. (81) constructed CAR-T cells targeting CD276. They found that CD276 CAR T cells control tumor growth *in vitro* and *in vivo*. CD276 CAR-T showed stronger anti-tumor activity when targeted at tumor cells expressing PD-L1. Du et al. believed that CAR T cells significantly inhibit tumor growth without obvious toxicity or side effects. These evidence all support the prospect of CD276 CAR-T cell therapy being promising in patients. CAR-T cell therapy has shown great success in the treatment of recurrent childhood acute lymphocytic leukemia. However, due to the lack of reliable, targeted, and differentially expressed molecular cell surface markers in solid tumors, CAR-T cell technology has not yet been applied. Majzner et al. (82) developed a new CD276 CAR-T cell which binds to a monoclonal antibody. The antibody preferentially binds to tumor tissues and safety has been tested in clinical trials. By detecting CD276 CAR-T cells in a variety of childhood cancer models, Majzner et al. found that CD276 CAR-T cells exhibit anti-tumor activity *in vivo*, leading to regression of solid tumors in xenograft models including osteosarcoma, medulloblastoma, and Ewing’s sarcoma. They also demonstrated that the efficacy of CD276 CAR-T cells depended on the density of target antigens on the tumor surface, while the activity of cells expressing low levels of antigens is significantly reduced. In a recent study, Johanna et al. (83) found CD276 CAR T cells were highly active against atypical teratoid/rhabdoid tumors, for which most current therapies have been proven to be ineffective. In summary, increased expression level of CD276 in tumor tissues compared to normal tissues provides the potential for CD276 CAR-T cells as a treatment for tumors.

## Conclusion

The functionality of CD276 is complicated. Although great progress has been made in the past two decades, further research is still needed. The regulation of CD276 expression is closely related to tumor growth, invasion, metastasis, and immune escape. Due to increased expression levels of CD276 in multiple tumors and low expression in a variety of normal tissues, numerous studies used CD276 as the target for tumor gene therapy and monoclonal antibody therapy. Overall, CD276 does have great potential as an immunotherapeutic target, but its clinical value must be confirmed by further studies and clinical trials.

## Author Contributions

Conception and design: XW and YL. Acquisition of data: SL and JL. Analysis and interpretation of data: ZL, CZha, YW, and CZho. Drafting of the manuscript: SL and JL. Critical revision of the manuscript for important intellectual content: XW and YL. Administrative, technical, or material support: AW, FZ, KW, and FXZ. Supervision: XW and YL. All authors contributed to the article and approved the submitted version.

## Funding

This paper was supported by the following grants: China Scholarship Council 202006240301 to SL.

## Conflict of Interest

The authors declare that the research was conducted in the absence of any commercial or financial relationships that could be construed as a potential conflict of interest.
