# Supplementary figures and images for: The Role of CD276 in Cancers
Source: Front Oncol. 2021 Mar 26;11:654684. doi: 10.3389/fonc.2021.654684 (PMC8032984; doi:10.3389/fonc.2021.654684)

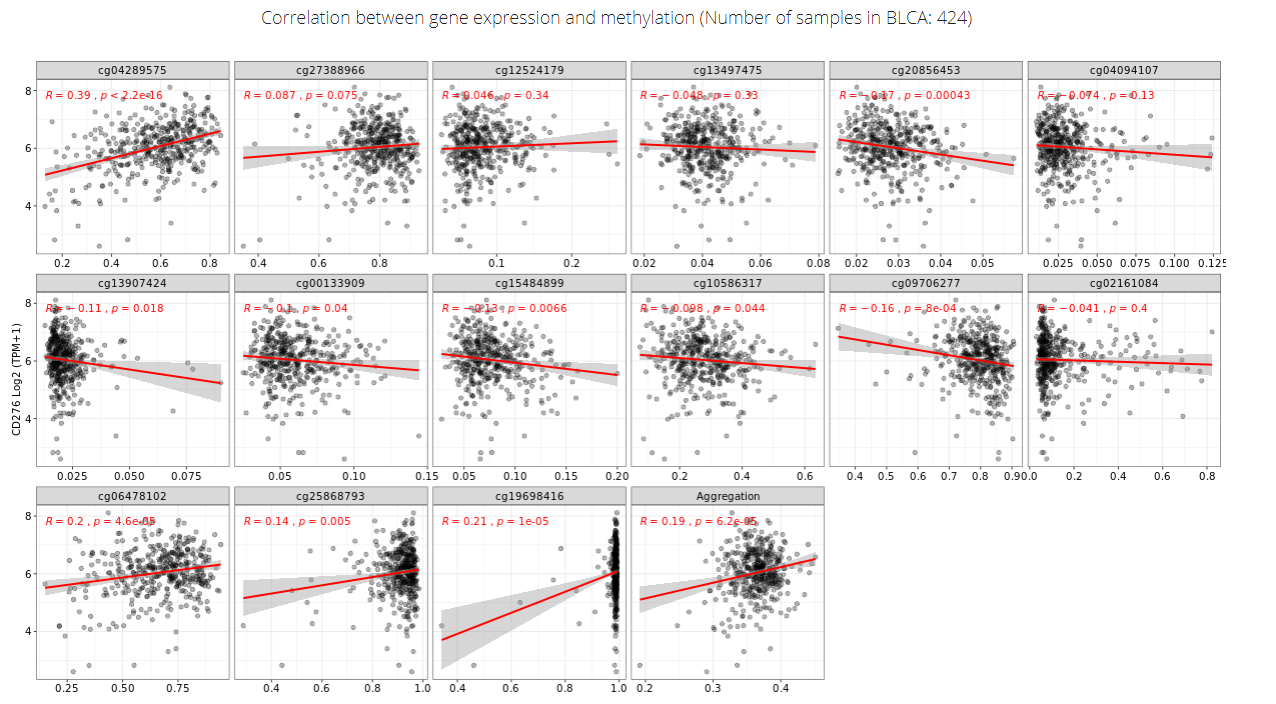

Supplement: Supplementary file 1 [file Image_1.png]

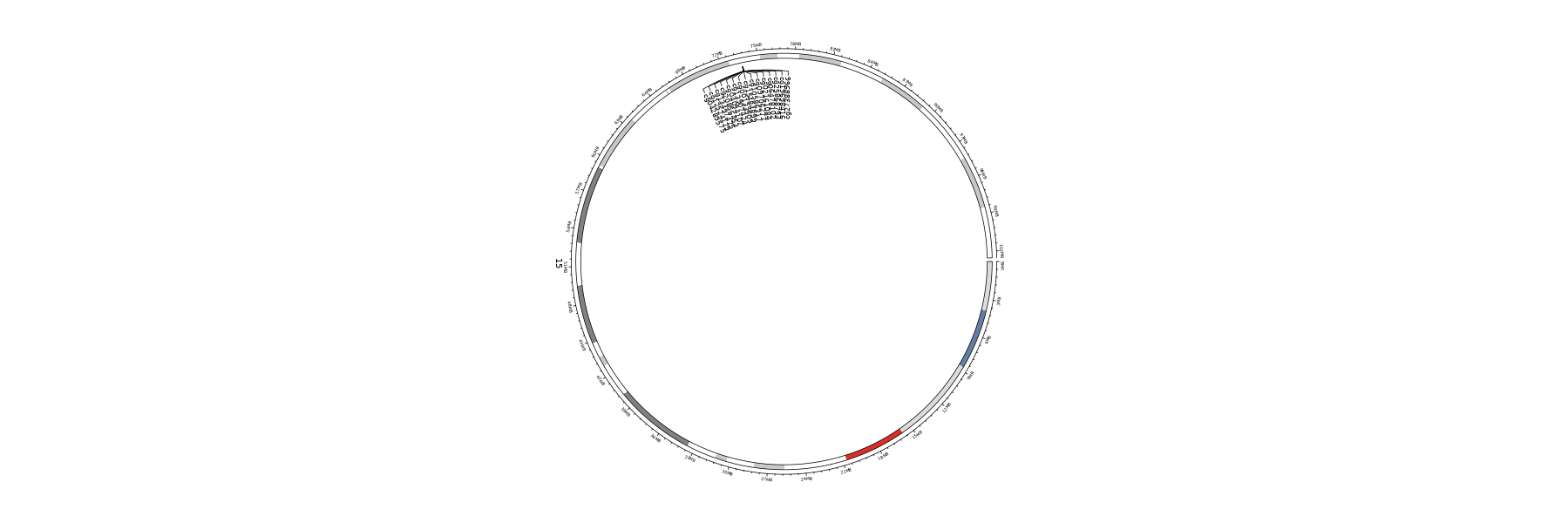

Supplement: Supplementary file 2 [file Image_2.png]

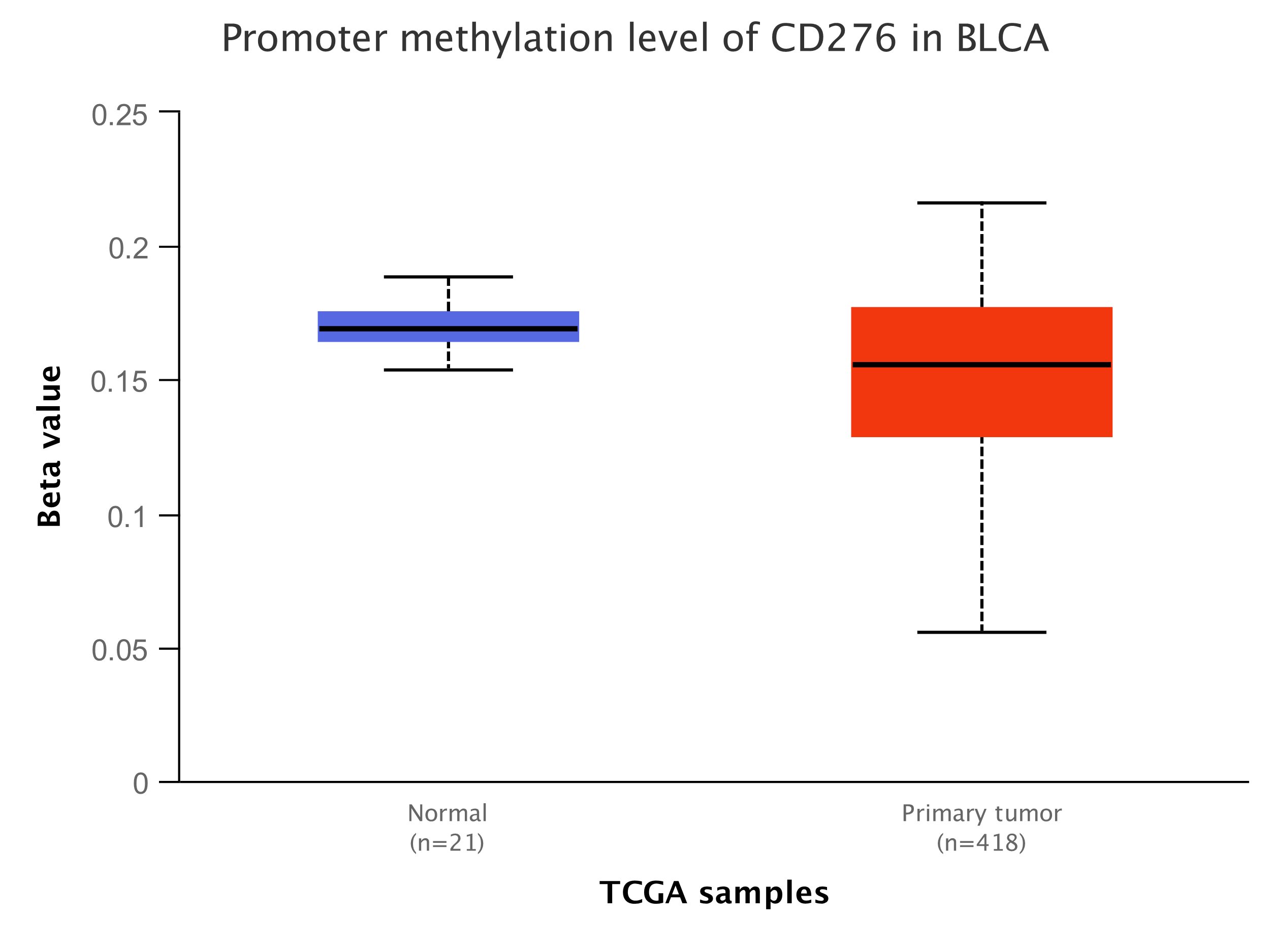

Supplement: Supplementary file 3 [file Image_3.jpeg]
